# Inhibition of Dopamine Transporter Activity by G Protein βγ Subunits

**DOI:** 10.1371/journal.pone.0059788

**Published:** 2013-03-26

**Authors:** Jennie Garcia-Olivares, Delany Torres-Salazar, William A. Owens, Tracy Baust, David P. Siderovski, Susan G. Amara, Jun Zhu, Lynette C. Daws, Gonzalo E. Torres

**Affiliations:** 1 Department of Neurobiology, University of Pittsburgh School of Medicine, Pittsburgh, Pennsylvania, United States of America; 2 Department of Pharmacology and Chemical Biology, University of Pittsburgh School of Medicine, Pittsburgh, Pennsylvania, United States of America; 3 Department of Physiology, University of Texas Health Science Center at San Antonio, San Antonio, Texas, United States of America; 4 Department of Pharmacology and UNC Neuroscience Center, The University of North Carolina at Chapel Hill, Chapel Hill, North Carolina, United States of America; 5 Department of Pharmaceutical and Biomedical Sciences, South Carolina College of Pharmacy, University of South Carolina, Columbia, South Carolina, United States of America; CNRS - Université Aix Marseille, France

## Abstract

Uptake through the Dopamine Transporter (DAT) is the primary mechanism of terminating dopamine signaling within the brain, thus playing an essential role in neuronal homeostasis. Deregulation of DAT function has been linked to several neurological and psychiatric disorders including ADHD, schizophrenia, Parkinson’s disease, and drug addiction. Over the last 15 years, several studies have revealed a plethora of mechanisms influencing the activity and cellular distribution of DAT; suggesting that fine-tuning of dopamine homeostasis occurs via an elaborate interplay of multiple pathways. Here, we show for the first time that the βγ subunits of G proteins regulate DAT activity. In heterologous cells and brain tissue, a physical association between Gβγ subunits and DAT was demonstrated by co-immunoprecipitation. Furthermore, *in vitro* pull-down assays using purified proteins established that this association occurs via a direct interaction between the intracellular carboxy-terminus of DAT and Gβγ. Functional assays performed in the presence of the non-hydrolyzable GTP analog GTP-γ-S, Gβγ subunit overexpression, or the Gβγ activator mSIRK all resulted in rapid inhibition of DAT activity in heterologous systems. Gβγ activation by mSIRK also inhibited dopamine uptake in brain synaptosomes and dopamine clearance from mouse striatum as measured by high-speed chronoamperometry *in vivo*. Gβγ subunits are intracellular signaling molecules that regulate a multitude of physiological processes through interactions with enzymes and ion channels. Our findings add neurotransmitter transporters to the growing list of molecules regulated by G-proteins and suggest a novel role for Gβγ signaling in the control of dopamine homeostasis.

## Introduction

Termination of dopamine (DA) neurotransmission is accomplished primarily through a plasma membrane sodium-dependent re-uptake process mediated by the dopamine transporter (DAT). The physiological contribution of DAT to the control of DA homeostasis has been suggested by decades of pharmacological studies and further substantiated by genetic approaches [1]. DAT gene deletion in mice leads to profound neurochemical changes characterized by a 95% decrease in total DA levels [2]. Indeed, depletion of DA stores in DAT knock-out mice was observed despite the fact that the DA synthesis rate was elevated by two-fold, suggesting a major role for the transporter in both the termination of DA signaling and vesicular DA replenishment.

Because DAT plays a critical role in the control of DA homeostasis, much attention has been placed on mechanisms regulating DAT activity and trafficking. Signaling pathways involved in rapid regulation of DAT include the activation of G protein-coupled receptors, intracellular second messenger systems, and the effect of protein-protein interactions [3]–[6]. As a consequence, these multiples modes of regulation result in dynamic changes in DAT function, which are expected to have profound consequences in DA transmission and DA-related behaviors.

We recently identified the synaptic vesicle protein synaptogyrin-3 as a DAT interacting protein [7]. The physical interaction between synaptogyrin-3 and DAT increases transporter activity and suggests a model in which the plasma membrane DA uptake process is physically and functionally coupled with vesicle monoamine transporter 2 (VMAT2)-mediated synaptic vesicle refilling. In this scenario, a macromolecular complex involving DAT, synaptogyrin-3, and VMAT2 would ensure a rapid and efficient transport of DA from the extracellular space into synaptic vesicles. Given the functional interaction between these two transporter systems, it is then tempting to speculate that DAT and VMAT2 might be regulated through similar signaling pathways.

VMAT2-mediated DA transport into synaptic vesicles has been shown to be regulated by G proteins [8]. Specifically, the synaptic vesicle-associated G protein Gαo_2_ decreases VMAT2-mediated vesicular DA uptake, although it is not known whether this effect is the result of a direct interaction between the vesicular transporter and the G protein. Interestingly, the reduction of VMAT2 activity by G proteins was dependent on the vesicular content of monoamines [9]. Site-directed mutagenesis identified the first luminal domain of VMAT2 as being responsible for the G protein down-regulation, leading the authors to propose this domain as a monoamine level sensor regulated by Gαo_2_ subunits. Because G subunits are associated with synaptic vesicles [10], this mode of regulation appears to be G protein-coupled receptor-independent.

Motivated by these provocative findings describing a functional interaction between VMAT2 and synaptic vesicle-associated G proteins, we examined the possibility that DAT-mediated uptake might also be regulated by G proteins. In the present study, we identify a novel interaction between DAT and Gβγ subunits that result in modulation of DAT activity.

## Materials and Methods

### Cell Culture and Transfections

HEK293 cells were obtained from the American Type Culture Collection. HEK293 cells were cultured in MEM supplemented with 10% fetal bovine serum (FBS), 1 mM glutamine, and 50 µg/ml each penicillin and streptomycin at 37°C in a humidified, 5% CO_2_ incubator. MN9D cells were provided by Dr. Alfred Heller (University of Chicago, Chicago, IL) and maintained in DMEM high glucose, supplemented with 10% FBS, at 37°C in a humidified, 5% CO_2_ incubator. The human DAT cDNA was cloned into pcDNA3.1(+) using *Kpn*I and *Xba*I sites and used to transfect HEK293 or MN9D cells with Lipofectamine 2000 (Invitrogen) combined with a CombiTag Magneto transfection reagent (OZ Biosciences). DAT-expressing single clones (HEK293-DAT or MN9D-DAT cells) were selected with G418 (Gibco), verified by DAT immunoblot and immunofluorescence, and maintained in appropriate media containing 0.5 mg/ml G418. Cells were transiently transfected using Lipofectamine 2000 (Invitrogen).

### Immunoprecipitations and Western Blot Analysis

C57BL/6 mice were purchased from Jackson Laboratories, whereas DAT knockout mice tissue was a generous gift from Dr. Marc Caron (Duke University). All procedures were carried out in accordance with the National Institute of Health's Guide to the Care and Use of Laboratory Animals, and were approved by the University of Pittsburgh’s Institutional Animal Care and Use Committee (IACUC) (Protocol number: IC-IS00000171-1) and in the case of DAT knockout mice brain samples, procedures were approved by the Duke University IACUC (Protocol number: A183-10-07). Striatum and cerebellum from wild-type and DAT knockout mice, HEK293-DAT, MN9D-DAT, or control cells were lysed in buffer containing (in mM): 20 HEPES, 125 NaCl, 1 EDTA, 1 EGTA and 10% glycerol containing protease inhibitors. After homogenization, 1% Triton X-100 was added, incubated for 1 h at 4°C, and centrifuged at 12000×g for 15 min at 4°C to remove cellular debris. Protein concentration was determined using the Dc Protein Assay kit (Bio-Rad Laboratories). Immunoprecipitations were carried out using 1 mg of total protein as described previously [7]. Two different DAT antibodies were used for immunoprecipitations; DAT^1^ (Mab369, Millipore), DAT^2^ (H-80, Santa Cruz). Immunoblotting was performed with a third DAT antibody, DAT^3^ (c-20, Santa Cruz) and a Gβ pan-antibody (T-20, Santa Cruz).

### GST Fusion Protein Pull-down Assays, Western Blot, and Immuno-Far Western Blot

cDNA fragments coding for intracellular domains of DAT were amplified by PCR and subcloned into the pGEX4T-1 vector. Plasmids construction, sequencing, and characterization have been described previously [11]. We generated three Gluthatione-S-Transferase (GST) fusion proteins: (1) GST-DAT_N_ (amino acids 1 to 60); (2) GST-DAT_L_, (amino acids 119–139); and (3) GST-DAT_C_ (amino acids 582–620) of DAT. For pull-down experiments, 5–20 µg of GST fusion proteins were incubated with striatum lysates for 30 min at 22°C. GST fusion proteins and subsequent interacting proteins were isolated with 40 µl of Gluthatione-resin and samples were analyzed by SDS-PAGE and Western blotting. Direct interactions were examined by Immuno-Far Western. Briefly, 5 µg of GST fusion proteins were loaded into SDS-PAGE gels and electro-transferred to nitrocellulose membranes. Membranes were then washed, blocked overnight with PBS containing 1% BSA and 0.05% Tween-20, and incubated with purified Gβγ protein from bovine brain (EMD Biosciences) for 30 min. After washing membranes with 50 mM Tris, 150 mM NaCl, pH 8.0 four times, Western Blot was performed with specified antibodies.

### [^3^H]-DA and [^3^H]-Glutamate Uptake in *Xenopus laevis* Oocytes

Capped RNAs (cRNA) encoding human DAT or human excitatory amino acid transporter 1 (EAAT1) were synthesized from SmalI-linearized pOTV-hDAT or pOTV-hEAAT1 using a MESSAGE machine kit (Ambion). Synthesized cRNA was resuspended in 10 µl of water and stored in 2 µl aliquots at −80°C until use. 50 nl of cRNA was injected into *Xenopus laevis* oocytes using a nanoliter injector (nanoliter 2000, World Precision Instruments), and oocytes were kept at 18°C in ND-96 buffer (in mM: 96 NaCl, 4 KCl, 0.3 CaCl2, 1 MgCl2 and 5 Hepes, pH 7.4) supplemented with 2.5 mM sodium pyruvate and 100 µg/ml gentamycin sulfate. Experiments were performed 2–3 days after cRNA injection. Intracellular injections of 50 nl of 100 µM GDP-β-S or GTP-γ-S (Sigma-Aldrich) were performed 15 min before the uptake experiment. Control experiments were performed with intracellular injections of H_2_O. After treatment with GTP analogs, oocytes were incubated for 10 min in 1 ml of ND-96 buffer containing 0.2 µM of [^3^H]-DA and 9.8 µM of DA or 0.2 µM of [^3^H]-Glutamate (PerkinElmer). Oocytes were transferred to non-radioactive ND-96 buffer, and washed three more times with ice-cold stop solution. Individual oocytes were lysed with 1 ml of 1% SDS for at least 1 h before adding the scintillation counting solution. For experiments with the mSIRK peptide, oocytes expressing DAT or EAAT1 were incubated for 30 min with ND-96 solution containing 10 µM of the peptide or 0.1% DMSO as control.

### [^3^H]-DA Uptake Assay in Cell Lines

The conditions to examine DAT-mediated uptake in cultured cells have been described previously [12]. Briefly, 72–96 h after transfections, medium was removed, and DAT-mediated uptake was measured after incubation of cells for 5 min with 250 µl of uptake buffer (in mM: 5 Tris base, 7.5 HEPES, 120 NaCl, 5.4 KCl, 1.2 CaCl_2_, 1.2 MgSO_4_, 1 ascorbic acid, and 5 glucose, pH 7.4). For HEK293-DAT cells, 20 nM of [^3^H]DA (3,4-[7-^3^H] dihydroxyphenylethylamine) (34.8 Ci/mmol; PerkinElmer) and increasing concentrations of cold DA ranging from 0.1 µM to 30 µM were used. After rinsing with 1 ml of NaCl-free uptake buffer, cells were solubilized in 0.5 ml of 1% SDS and the radioactivity incorporated into the cells was measured by liquid scintillation counting. Nonspecific uptake was determined in the presence of 300 µM cold DA. Data are presented as the mean ± SE. For experiments with the impermeable GTP analog GTP-γ-S, HEK293-DAT cells were permeabilized with streptolysin-o (SLO, Sigma) according to the method described in [13] with some modifications. Briefly, cells were incubated for 15 min at 37°C in Hank’s Balance Salt solution (HBSS) (in mM: 137 NaCl, 5.4 KCl, 0.25 Na_2_HPO_4_, 0.44 KH_2_PO_4_, 1.0 MgSO_4_, 4.2 NaHCO_3_, 5 Glucose, and 30 HEPES, pH 7.2). Cells were incubated for 20 min at 37°C with 100 ng/ml SLO containing 50 µM GTP-γ-S and 1 mM Dithiotrietol, followed by incubation with ice-cold HBSS containing 1.4 mM CaCl_2_ and 30 mM HEPES for 2 h. In other experiments, cells were incubated with the mSIRK peptide (myr-SIRKALNILGYPDYD) (EMD Chemicals) or the scramble version (scb-mSIRK) (myr-SLYRLISLAPRGDYD) (NeoBioScience) prior to uptake. In these experiments, control cells were incubated with 0.1% DMSO. Uptake was normalized to protein concentrations determined using the D_c_ protein assay kit.

### Biotinylation Assay

Transfected cells were washed three times with PBS and then incubated with gentle agitation for 30 min at 4°C with 1 ml of 1.5 mg/ml sulfo-NHS-SS-biotin prepared in Biotinylation buffer (in mM: 150 NaCl, 2 CaCl_2_, 10 triethanolamine, pH 7.8). The reaction was quenched by incubating the cells for an additional 10 min with 50 mM glycine in PBS. Cells were then washed with PBS and incubated in radioimmune precipitation assay buffer (RIPA) (in mM, 10 Tris, 150 NaCl, 1 EDTA, 0.1% SDS, 1% Triton X-100, and 1% sodium deoxycholate, pH 7.4) at 4°C for 1 h. Each sample was divided into two aliquots. One aliquot was used for isolation of biotinylated proteins with ultralink-immobilized avidin beads (Pierce). The second aliquot was used to determine total DAT levels. Samples were analyzed by SDS-PAGE and Western blotting with the anti-DAT antibody (MAB369, Millipore) and an HRP-conjugated secondary antibody (Jackson Immunoresearch Lab). Densitometry analysis of bands was performed with ImageJ software (U.S. National Institutes of Health).

### [^3^H]-DA Uptake in Synaptosomes

Synaptosomes were prepared from rat striata as described [14]. Striatal synaptosomes were preincubated with various concentrations of mSIRK or scb-mSIRK (0.01 µM-100 µM) at 34°C for 10 min followed by the addition of [^3^H]-DA (final concentration, 0.1 µM) for 8 min. Data are expressed as percentage of control values (20205±2065 dpm). Nonspecific [^3^H]-DA uptake was determined in the presence of 10 µM nomifensine. Kinetic analysis of the synaptosomal [^3^H]-DA uptake was determined in the absence (control) or presence of 5 µM mSIRK. Synaptosomes were preincubated with or without mSIRK at 34°C for 10 min followed by the addition of one of eight mixed concentrations of the [^3^H]-DA. In parallel, nonspecific uptake at each concentration of [^3^H]-DA in the presence of 10 µM nomifensine, was subtracted from total uptake to calculate DAT-mediated uptake.

### *In vivo* Electrochemical Recordings of Striatal DA Clearance

Clearance of exogenously applied DA from the striatum of anesthetized male C57Bl/6 mice was measured by high-speed chronoamperometry using the FAST-12 system (Quanteon) as previously described [15]. All procedures involving the use of mice were approved by the University of Texas Health Science Center at San Antonio IACUC (Protocol number 02014×). Carbon fiber electrodes were coated with Nafion® (Aldrich Chemical Company). Neither peptide (mSIRK nor scrambled) elicited an electrochemical signal *in vitro* or *in vivo*. The center-to-center distance between the microelectrode and the micropipette ejector was ∼200 µm. The micropipette was filled with DA (200 µM), mSIRK, or scrambled peptide (both 100 µM). The electrode/micropipette assembly was lowered into the striatum (in mm from bregma: A/P, +1.1; M/L, ±1.4; D/V, −2.25). Drug application was accomplished using a Picospritzer II (Parker Hannifin Corporation) in an ejection volume of ∼20 nl for DA to deliver ∼4 pmol and attain signals at the recording electrode in the range of 0.5 to 1.0 µM, and 50 nl for peptides to deliver 5 pmol (5–25 psi for 0.25–3 s) with an estimated concentration of peptide reaching the recording electrode in the range of 0.5–10 µM [16]. Oxidation potentials consisting of 100 ms pulses of 550 mV each separated by a 1 s interval during which the resting potential was maintained at 0 mV were applied to the microelectrode with respect to an Ag/AgCl reference electrode implanted into the contralateral superficial cortex. Oxidation and reduction currents were digitally integrated during the last 80 ms of each 100 ms voltage pulse. For each recording session, DA was pressure-ejected at 5 min intervals until reproducible signals were obtained.

### Data Analysis

Functional experiments and densitometry data were analyzed with SigmaPlot (Systat Software Incorporation). Data are presented as the mean of at least three independent experiments with the standard error of the mean (S.E.M.) value. For uptake experiments, the V_max_ and K_m_ values were estimated by fitting the data to the Michaelis-Menten equation and represent the means from three independent experiments ± S.E.M. Statistics for uptake experiments were performed using a non-paired t-test with an accepted significance level at p<0.05. *In vivo* clearance was analyzed with ANOVA followed by Bonferroni or Newman-Keuls post-hoc comparisons.

## Results

### Physical Interaction between DAT and Gβγ Subunits

**Figure 1:**
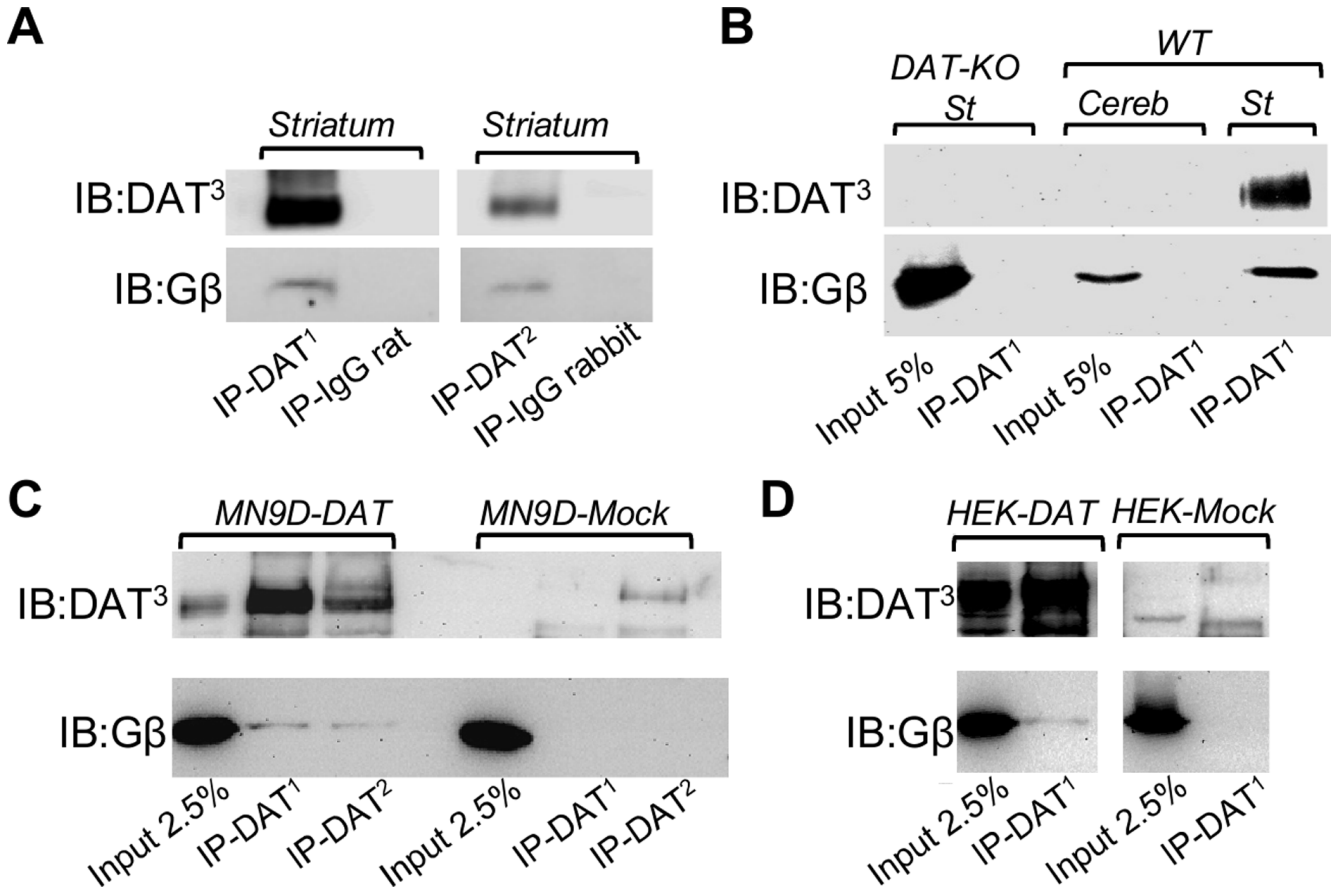
Co-immunoprecipitation of DAT and Gβγ subunits. (**A, B**) Immunoprecipitation of DAT with two DAT antibodies directed against different DAT epitopes results in the co-precipitation of Gβ subunits from mouse striatum. Control experiments include immunoprecipitations with nonspecific rat or rabbit IgGs or immunoprecipitations from DAT knockout striatum or mouse cerebellum. (**C, D**) Immunoprecipitation of DAT resulted in the co-immunoprecipitation of Gβ subunits from MN9D-DAT cells or HEK293-DAT cells. Control experiments include immunoprecipitations from MN9D or HEK293 mock-transfected cells. Three different DAT antibodies were used; DAT^1^ (Mab369, Millipore), DAT^2^ (H-80, Santa Cruz), and DAT^3^ (c-20, Santa Cruz). Immunoblotting was performed with a Gβ pan-antibody (T-20, Santa Cruz). Cereb = cerebellum, St = striatum, DAT-KO = DAT knockout.

**Figure 2:**
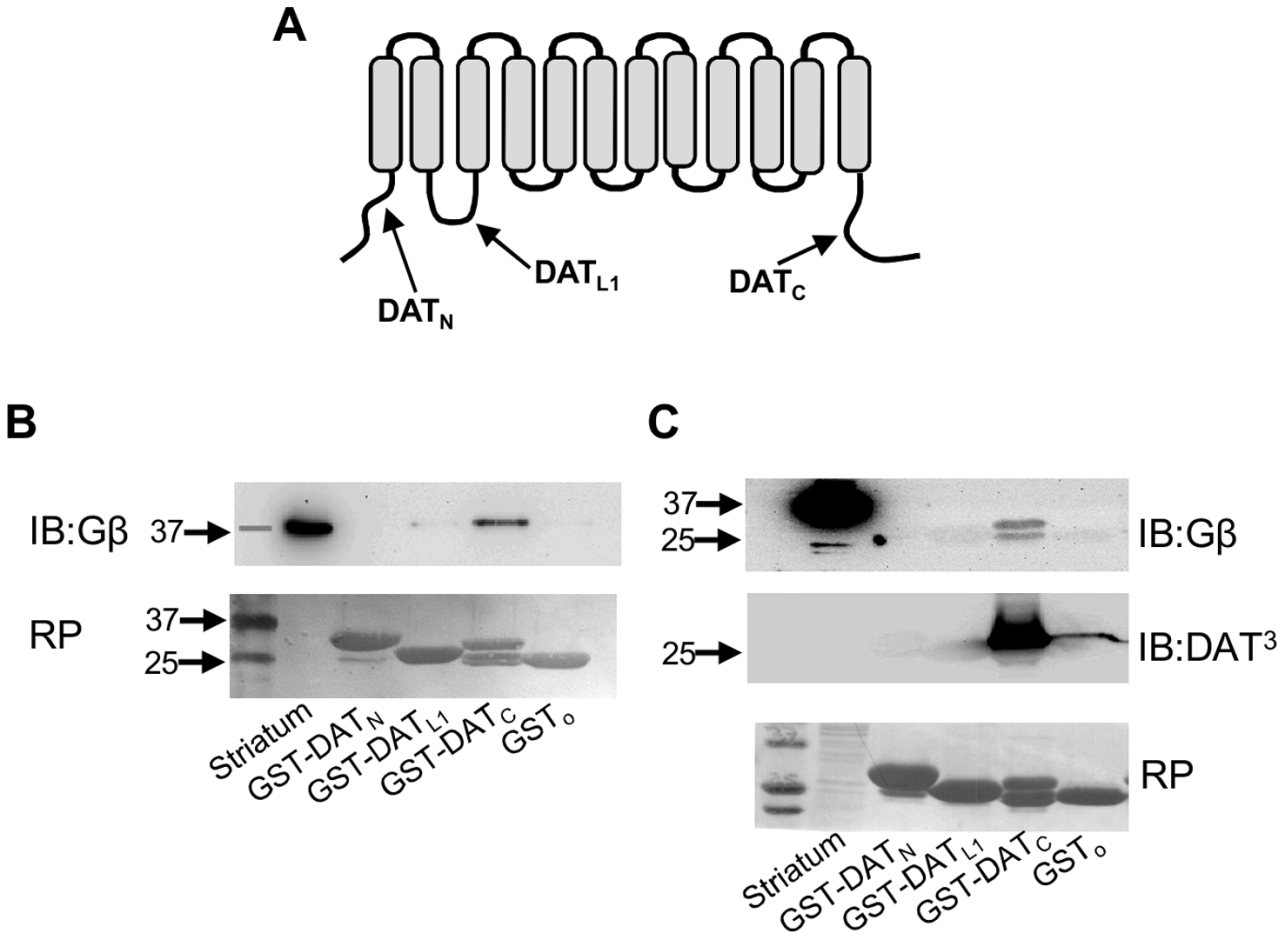
Direct interaction between Gβγ subunits and the carboxy terminus of DAT. (**A**) Schematic representation of DAT depicting cytoplasmic segments used in GST pull-down assays. (**B**) The carboxy terminus of DAT (GST-DAT_c_) precipitated Gβγ subunits from mouse striata. (**C**) Immuno-far-western assay showing a direct interaction between GST-DAT_c_ and purified Gβγ from bovine brain. RP = Ponceau Red.

To begin to investigate the possibility that G proteins regulate DAT activity, we first examined whether there is a physical association between the transporter and the subunits of G proteins. We generated three lines of evidence supporting a physical interaction between DAT and G protein βγ subunits. First, immunoprecipitations with two antibodies directed against different DAT epitopes resulted in co-precipitation of Gβγ subunits from mouse striatum, while no bands were detected in samples precipitated using the corresponding control IgGs (Figure 1A). In mouse cerebellum or striatal tissue from DAT knockout mice; where DAT expression is not detectable, immunoprecipitation with DAT antibodies failed to co-precipitate Gβγ subunits (Figure 1B), confirming the specificity of our DAT antibodies. Furthermore, the DAT-Gβγ interaction was recapitulated in heterologous cells lines (MN9D and HEK293) stably expressing human DAT (Figure 1C–D). We failed to detect any interaction with Gα using a pan-antibody in co-immunoprecipitation experiments (data not shown). While these results cannot rule out the possibility of an interaction between DAT and Gα subunits, they strongly support a physical association between the transporter and Gβγ subunits. Secondly, to identify the DAT domains involved in the interaction with Gβγ subunits, we generated and purified three GST fusion proteins containing the amino-terminus, the first intracellular loop, or the carboxy-terminus of DAT (Figure 2A) and performed pull-down assays. Only the carboxy-terminus of DAT was able to pull down Gβγ from mouse striatal lysates (Figure 2B). Finally, a direct interaction between the carboxy-terminus of DAT and Gβγ subunits was demonstrated by Immuno-Far Western using commercially available Gβγ subunits purified from bovine brain (Figure 2C). Thus, these results demonstrate a direct physical interaction between DAT and Gβγ subunits and identify the carboxy terminus of the transporter as the site of the interaction.

### Activation of G Proteins by GTP-γ-S Inhibit DAT Activity in Heterologous Systems

**Figure 3:**
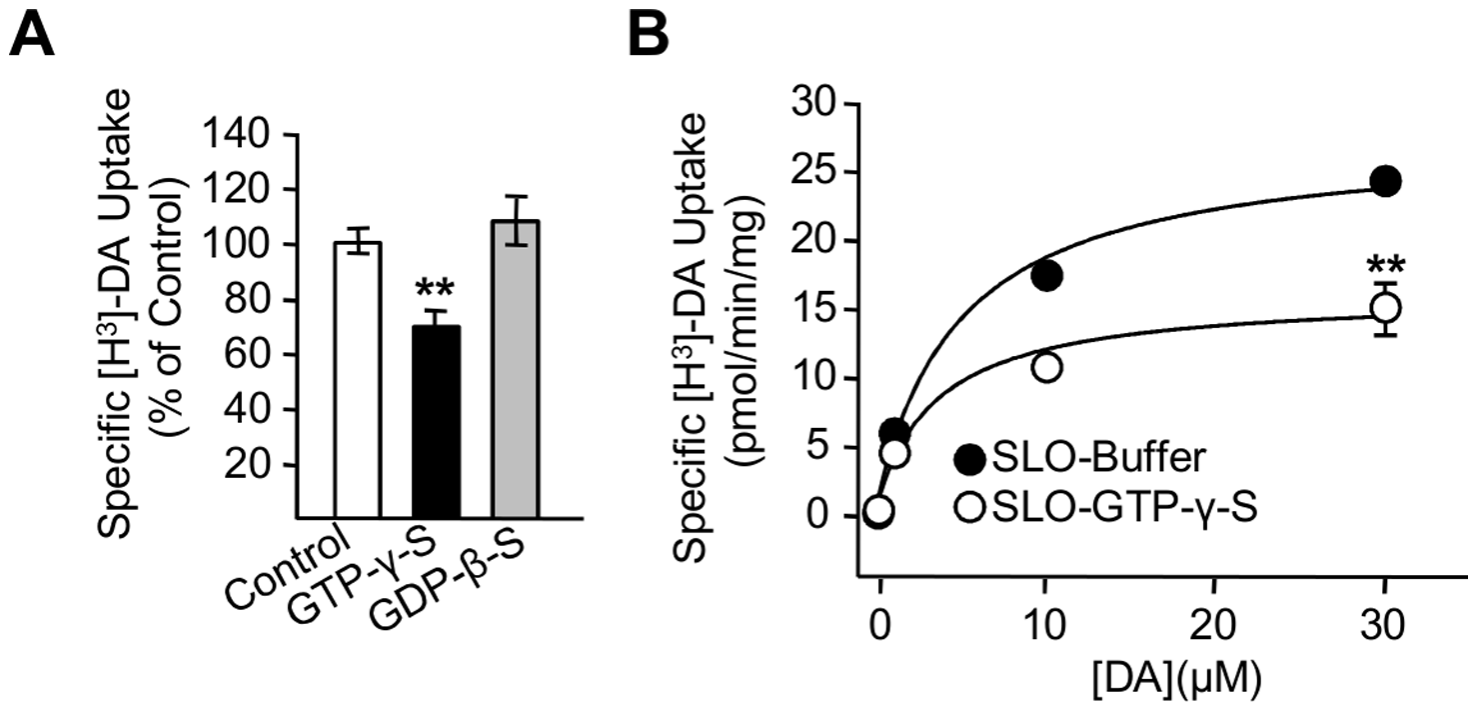
Activation of G proteins with GTP-γ-S inhibits DAT function in heterologous systems. (**A**) [^3^H]-DA uptake assays in *Xenopus laevis* oocytes expressing DAT after intracellular injection with water (white bar, n = 8), 10 µM GTP-γ-S (black bar, n = 8), or 10 µM GDP-β-S (gray bar, n = 8). (**B**) Specific [^3^H]-DA uptake and kinetic analysis for HEK293-DAT permeabilized with streptolysin-O (SLO) and dialyzed with 50 µM GTP-γ-S (white circles, n = 3). or control buffer (black circles, n = 3). **p<0.01.

According to the classical model, G proteins are activated when GTP binds to the Gα subunit of the Gαβγ trimeric complex, resulting in the dissociation of the Gα from the Gβγ dimer. Subsequent hydrolysis of GTP into GDP allows the Gα and Gβγ subunits to re-associate, consequently inactivating the Gαβγ complex [17]. Based on this model, we employed various pharmacological approaches to examine the effects of G protein activation on DA uptake. The non-hydrolyzable GTP analog GTP-γ-S binds to Gα and activates G proteins by dissociating Gα subunits from Gβγ dimers. Because GTP-γ-S is cell impermeable, we employed *Xenopus laevis* oocytes expressing human DAT where the GTP analog can be injected directly into the cytoplasm. Injection of GTP-γ-S (10 µM) resulted in 30.5±6.1% reduction of [H^3^]-DA uptake in *Xenopus laevis* oocytes expressing human DAT (Figure 3A). In contrast, intracellular injection of GDP-β-S (10 µM), which prevents Gαβγ complex dissociation, failed to alter DAT uptake (Figure 3A). We repeated these experiments in HEK293-DAT cells permeabilized with streptolysin prior to incubation with the GTP analog GTP-γ-S. As shown in Figure 3B, GTP-γ-S incubation produced a significant decrease in DAT uptake activity, consistent with the results obtained in oocytes. Thus, these findings suggest that the activation of endogenous G proteins in both, *Xenopus* oocytes and HEK293 cells expressing DAT results in inhibition of transporter activity.

### Overexpression of Gβγ Subunits Inhibit DAT Activity in Heterologous Cells

**Figure 4:**
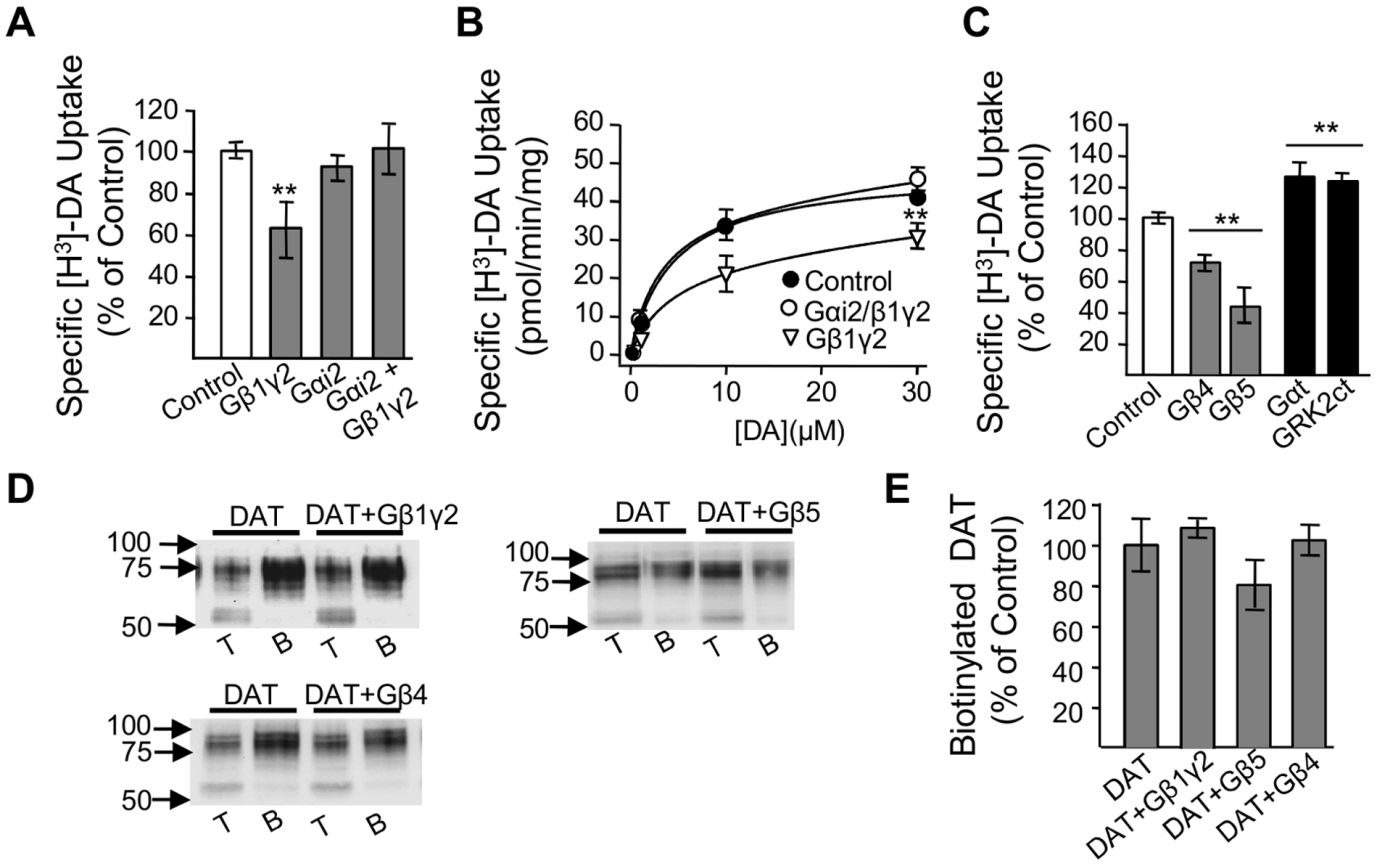
Overexpression of Gβγ subunits results in decreased DAT uptake activity in HEK-293-DAT cells. (**A, B**) Specific [^3^H]-DA uptake and kinetic analysis for HEK293-DAT control cells (white bar, n = 6) or transfected with Gβ1γ2 (n = 3), Gα_i2_ (n = 3), or Gα_i2_Gβ1γ2 (n = 3). (**C**) Overexpression of Gβ4 (n = 5) or Gβ5 (n = 7) (gray bars), decreased [^3^H]-DA uptake in HEK293-DAT cells when compared to control (white bar, n = 19). Overexpression of the Gβγ scavengers (black bars), Gα_t_ (n = 6), or GRK2ct (n = 4) increased [^3^H]-DA uptake. (**D, E**) Overexpression of Gβ1γ2 (n = 4), Gβ4 (n = 5), or Gβ5 (n = 7) in HEK-DAT cells did not alter the plasma membrane levels of DAT as measured by biotinylation. T = total fraction, B = biotinylated fractions, **p<0.01.

As a second functional approach, we examined the effect of overexpressing Gβγ subunits on DAT activity in HEK293 cells stably transfected with DAT. Overexpression of Gβ1γ2, the most common Gβγ dimer expressed in brain, resulted in a 37.1±13.8% reduction in [H^3^]-DA uptake (Figure 4A). In an effort to show that the inhibitory effect mediated by Gβγ subunits is reversible, we repeated the experiments in the presence of a Gα subunit. Overexpression of Gα_i2_ failed to alter DAT activity, but more importantly prevented the inhibitory effect of Gβ1γ2 on DAT-mediated uptake (Figure 4A). The changes in DAT function by Gβ1γ2 overexpression were the result of a reduction in the maximal velocity (V_max_), but not in the affinity (K_m_) of the transporter for DA (Figure 4B). There are five distinct Gβ isoforms, all expressed in brain. Therefore, to investigate the possibility that the inhibitory effect observed by Gβ1γ2 is isoform specific, we repeated the experiments using the most divergent Gβ4 or Gβ5 subunits. Overexpression of Gβ4 or Gβ5 either alone or in combination with γ2 subunits produced similar decreases in DAT activity in HEK293 cells (Figure 4C) suggesting that the effect is not isoform specific. Biotinylation experiments revealed that Gβ5, Gβ4, or Gβ1γ2 overexpression resulted in no differences in cell surface levels of the transporter compared to control cells (Figure 4D and 4E). Thus, these results indicate that the decrease in DAT uptake activity by Gβγ subunits is not a consequence of decreased plasma membrane levels of the transporter.

### Sequestration of Endogenous Gβγ Subunits Increase DAT activity in Heterologous Cells

Next, we examined the involvement of endogenous Gβγ subunits on DAT modulation by transfecting HEK293-DAT with either Gα_transducin_ (Gα_t_) or the carboxy-terminal domain of G protein–coupled receptor kinase 2 (*GRK2ct*), which are both known scavengers of Gβγ subunits [18]–[19]. Overexpression of Gα_t_ or GRK2ct significantly increased DAT activity (126.3%±9.1% and 123.2%±6.7%, respectively) (Figure 4C). These results suggest that DAT is tonically inhibited by Gβγ subunits expressed in HEK293 cells.

### Activation of Endogenous Gβγ Subunits with mSIRK Inhibit DAT Activity in Heterologous Systems

**Figure 5:**
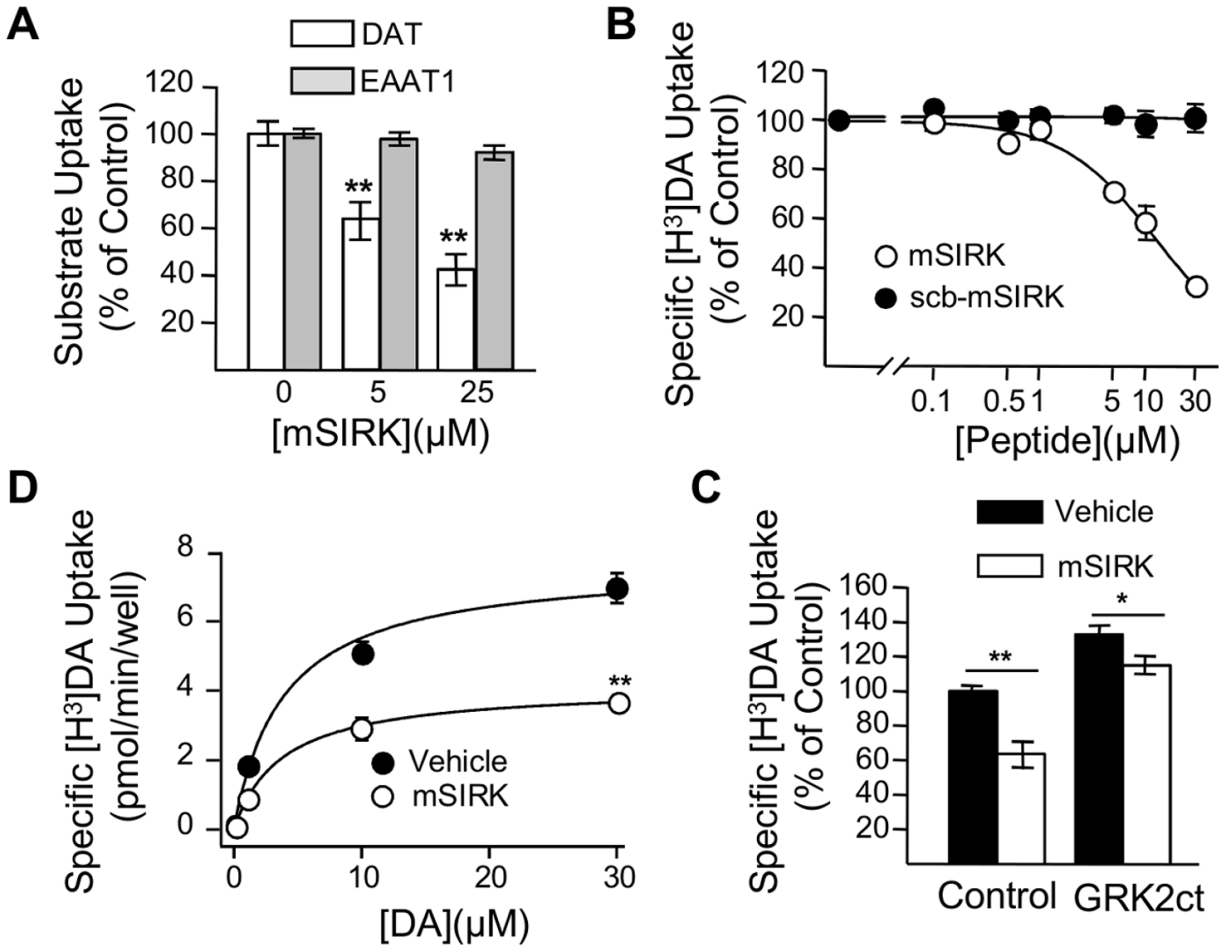
Activation of Gβγ with mSIRK resulted in an inhibition of DAT activity in heterologous systems. (**A**) Oocytes expressing DAT (white bars) or EAAT1 (gray bars) were incubated for 30 min with 0.5% DMSO (DAT/n = 3; EAAT1/n = 25), 5 µM mSIRK (DAT/n = 20, EAAT1/n = 23), or 25 µM mSIRK (DAT/n = 15, EAAT1/n = 15) before uptake assays with [H^3^]-DA or [^3^H]-Glutamate, respectively. (**B**) Dose-dependent inhibition of [^3^H]-DA uptake in HEK293-DAT cells by mSIRK (n = 4, white circle). (**C**) 5 min pre-incubation of HEK293-DAT cells with 10 µM mSIRK produced a reduction of V_max_ with no changes in K_m_ (n = 3). (**D**) In HEK293-DAT, 10 µM mSIRK reduced uptake (control, black bar, n = 32; mSIRK, white bar, n = 15). The inhibitory effect of 10 µM mSIRK was attenuated in HEK-DAT cells expressing GRK2ct (control, n = 16; mSIRK, n = 16). **p<0.01 or *p<0.05.

As an additional approach to assess the role of Gβγ on DAT function, we tested the effect of mSIRK, a cell-permeable myristoylated peptide that specifically activates Gβγ subunits [20]–[21]. In *Xenopus laevis* oocytes expressing DAT, incubation with 5 µM or 25 µM of mSIRK resulted in a 34.0±8.0% and 57.3±6.3% decrease in DAT activity, respectively (Figure 5A). To examine specificity, we tested the effect of mSIRK on glutamate uptake in oocytes expressing the excitatory amino acid transporter 1 (EAAT1). Here, glutamate uptake was not affected by mSIRK under the same conditions (Figure 5A). Next, we repeated these experiments in HEK293-DAT cells. mSIRK pre-incubation for 5 min produced a dose-dependent inhibition of [H^3^]-DA uptake with an IC_50_ of 10.6±2.6 µM; whereas, a scramble myristoylated peptide (scb-mSIRK) failed to alter DA uptake (Figure 5B). Longer incubation times with mSIRK did not augment the inhibitory effect on DAT function (IC_50_ = 13.2±1.2 µM). Kinetic analysis revealed that mSIRK incubation resulted in a reduction of V_max_ without changes in the K_m_ of DAT (Figure 5C)**.** To confirm that the effect of mSIRK is mediated by Gβγ subunits, we repeated these experiments in HEK293-DAT cells transfected with the Gβγ scavenger GRK2ct. Consistent with our previous results (Figure 4C), overexpression of GRK2ct in HEK293-DAT cells increased DAT activity. More importantly, the inhibitory effect of mSIRK was significantly attenuated in the presence of GRK2ct (Figure 5D). Thus, these results are consistent with those obtained with GTP-γ-S and Gβγ subunit overexpression and further support the contention that activation of Gβγ subunits results in inhibition of DAT activity.

### Activation of Endogenous Gβγ Subunits with mSIRK Inhibit DAT Activity in Striatal Synaptosomes

**Figure 6:**
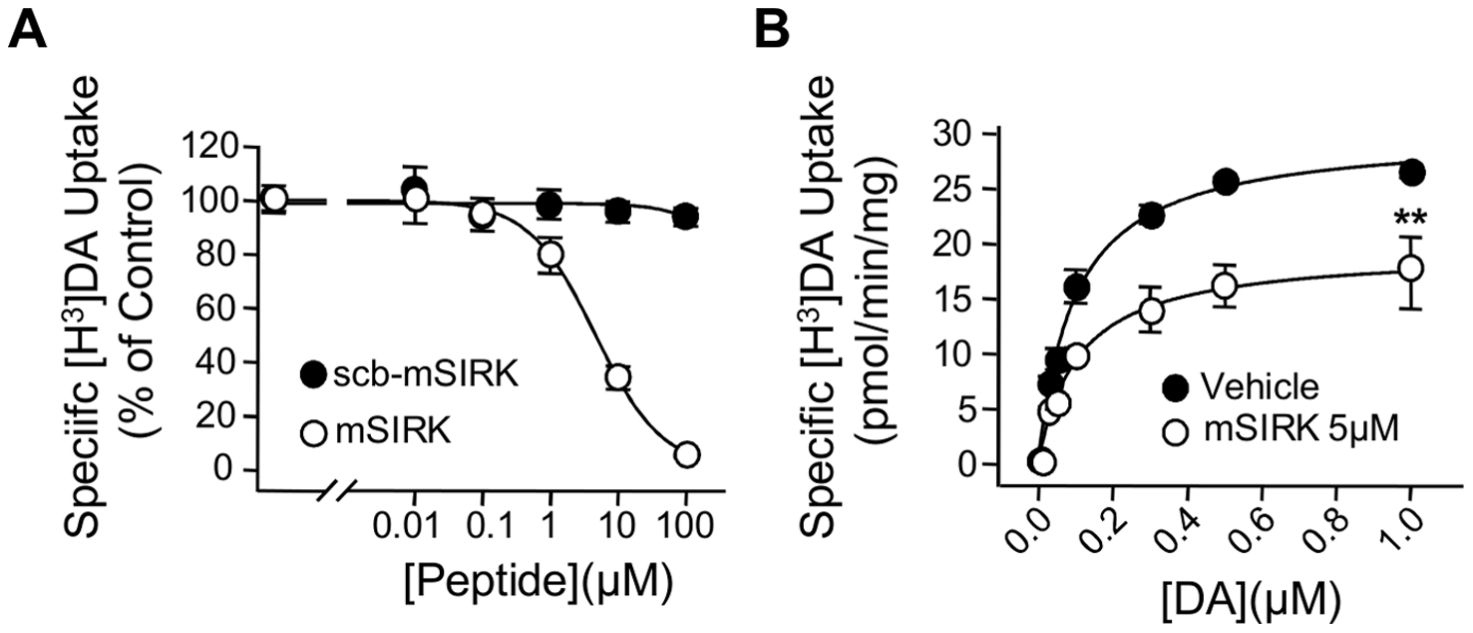
Activation of Gβγ subunits reduces DAT activity in brain synaptosomes (**A**) **mSIRK reduces [^3^H]-DA uptake in rat striatal synaptosomes.** Samples were pre-incubated for 10 min with various concentrations of mSIRK (open circles) or scb-mSIRK (filled circles) at 34°C, followed by the addition of [^3^H]-DA (final concentration, 0.1 µM) for 8 min, n = 3. Nonspecific [^3^H]-DA uptake was determined in the presence of 10 µM nomifensine. (**B**) Kinetic analysis of the synaptosomal [^3^H]-DA uptake was determined in the absence (vehicle) or presence of 5 µM mSIRK, n = 3. **p<0.01.

To examine the Gβγ-mediated regulation of DAT in native systems, we first assessed the effect of mSIRK on DAT activity in striatal synaptosomes. Pre-incubation of synaptosomes for 5 min with mSIRK, but not with scb-mSIRK, resulted in a dose-dependent inhibition of DAT activity with an IC_50_ of 5.4±0.9 µM (Figure 6A), which is consistent with reported IC_50_ values for mSIRK in other systems [20]–[22]. As observed with heterologous cells, the effect of mSIRK on DAT function reflected changes in V_max_ (37.7±4.6% reduction) without changes in K_m_ (Figure 6B).

### Activation of Endogenous Gβγ Subunits with mSIRK Inhibit DAT Activity *In Vivo*

**Figure 7:**
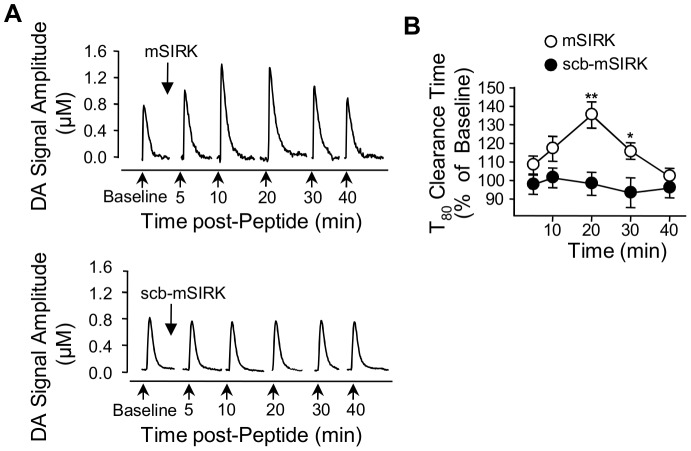
Activation of Gβγ subunits reduces DAT activity *in vivo.* (**A**) Clearance of exogenously applied DA from the striatum of anesthetized mice. Once a baseline for DA clearance was established, mSIRK or scr-mSIRK was applied, and then 5 min later, DA was applied and again, 10, 20, 30 and 40 min after the peptides were introduced (arrows). The amplitude of the DA signal augment as observed in a representative traces of DA signal after intrastriatal injection of mSIRK (upper panel) or scb-mSIRK (bottom panel). (**B**) Clearance time (T_80_) of exogenous DA was increased in the presence of mSIRK treatment. **p<0.01, *p<0.05.

Finally, we performed high-speed chronoamperometry to examine the *in vivo* effect of the mSIRK peptide on DA clearance in striatum. In these experiments, a recording microelectrode was placed in the striatum and a glass multi-barrel micropipette was positioned adjacent to the electrode to locally deliver DA, mSIRK, or scrambled peptide. For each recording session, DA was pressure-ejected at 5 min intervals before and after mSIRK or scrambled peptide. mSIRK application, but not scrambled peptide produced an increase in both the amplitude of the DA signal (Figure 7A) and the clearance time of extracellular DA (Figure 7B). This pattern is consistent with an inhibition of DAT uptake activity, which is typically observed with known inhibitors of DA transport [15]. Taken together, our findings from synaptosomal preparations and intact animals provide compelling evidence that G protein βγ subunits modulate DAT activity within a physiological context.

## Discussion

Here, we have used a combination of biochemical and functional approaches in heterologous systems, brain synaptosomes, and *in vivo* to demonstrate that Gβγ subunits regulate DAT activity. Biochemically, we provide evidence for a direct interaction between DAT and Gβγ through co-immunoprecipitation and pull-down assays with purified proteins. Functionally, Gβγ subunit activation by multiple approaches resulted in a rapid inhibition of transporter activity without alterations in levels of the transporter at the cell membrane. To our knowledge, no data are available describing an interaction between plasma membrane neurotransmitter transporters and G proteins and thus, this is the first study to identify a Gβγ-mediated modulation of a neurotransmitter transporter.

According to the traditional view, upon binding of an extracellular agonist to a G-protein coupled receptor (GPCR), G proteins dissociate into active Gα and Gβγ subunits. Originally, the Gα subunits were believed to transduce cellular responses, while the Gβγ subunits were regarded to function solely as negative regulators of Gα-mediated signaling. Investigations over the last 20 years have revealed that the activation and function of G proteins is far more complex than previously anticipated. There is now evidence for both, receptor-dependent and -independent Gα- and Gβγ-mediated responses [23]. In the case of VMAT2, the Gα-mediated inhibition of the transporter appears to be receptor-independent as Gα subunits are associated with synaptic vesicles [8], [10]. Our findings from immunoprecipitation experiments and those using Gβγ subunits scavengers suggest a receptor-independent regulation, however, we can’t rule out a receptor-mediated regulation of DAT activity through Gβγ subunits. Potential GPCRs expressed in DA neurons, which could modulate DAT activity include receptors for glutamate, norepinephrine, serotonin, ATP, and DA. Indeed, several studies have documented changes in DAT activity as a result of the activation of D2 DA receptors (D2R) [24]–[25]. Interestingly, recent provocative evidence suggests that the dopamine D2 receptor (D2R) regulates the trafficking of DAT to the plasma membrane through a direct protein-protein interaction [26]. Together, the available data suggest up-regulation of DAT through two mechanisms involving the activation of second messenger systems through D2R [25], as well as a direct protein-protein interaction between D2R and DAT [26]. Thus, it is unlikely that the Gβγ-dependent decrease in DAT activity is related to activation of D2R. Future studies will be required to examine whether the Gβγ-mediated inhibition of DAT described in this report is related to the activation of additional GPCRs or represents a receptor-independent mechanism.

Additionally, evidence has accumulated indicating that Gβγ subunits also transduce cellular signals. In fact, Gβγ subunits have been reported to directly regulate a diverse array of effector molecules including ion channels, enzymes, and intracellular regulators [27]. These interactions provide a role for Gβγ in important physiological functions such as cardiac membrane potential, heart rate, inflammation, pain modulation, and neurotransmitter release [17], [28]–[29]. Of the many identified Gβγ effectors, the Gβγ-mediated inhibition of voltage-dependent calcium channels has been one of the most widely studied and best characterized [30]. Gβγ directly binds and inhibits calcium channel activity. Likewise, Gβγ has also been reported to interact with syntaxin 1A [31], which is part of a SNARE protein complex that links the calcium channel to the vesicular release machinery. These findings suggest a complex interplay between the channel, G proteins, and SNARE proteins that modulate neurotransmitter release. Based on this model, we speculate that a similar network may exist between DAT, Gβγ, and synaptic vesicles. Indeed, we previously reported that DAT is also physically and functionally coupled to synaptic vesicles via an interaction with synaptogyrin-3 [7]. Further supporting this idea, syntaxin 1A has also been reported to interact and modulate DAT [32]–[34]. Future studies will be needed to explore this possibility.

Despite the fact that there is much to learn regarding the modulation of DAT by Gβγ subunits, the identification of DAT as a novel Gβγ effector will open new avenues to our understanding of DA homeostasis regulation. DAT plays a crucial role in the control of DA homeostasis and has been implicated in a variety of psychiatry disorders and drug addiction [35]. Our findings suggest a novel mechanism for the control of DA homeostasis and may introduce a novel target for treatment of DA-related disorders. It remains to be seen whether additional plasma membrane transporters such as the norepinephrine, serotonin, GABA, or glutamate transporters are also modulated by G proteins.
